# Supplementary material for: CortexCAM: A Camera Array Microscope for Cortex-Wide Cellular Imaging in Freely Locomoting Mice
Source: Res Sq. 2026 Jun 2:rs.3.rs-9005693. Preprint. [Version 1] doi: 10.21203/rs.3.rs-9005693/v1 (PMC13252553; doi:10.21203/rs.3.rs-9005693/v1)
Supplement: 1 [file nihpprs9005693v1-supplement-1.pdf]

Supplementary Information

a

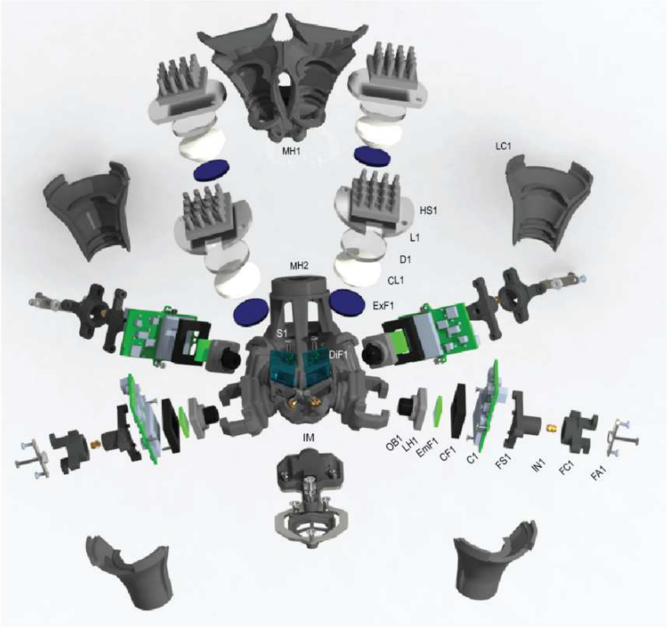

Bill of Materials

Index	Part ID	Description	Quantity (Nos)
1	OB1	Objective Lens	4
2	LH1	Lense Housing	4
3	EmF1	Emission Filter	4
4	CF1	Compressible Foam	4
5	C1	CMOS sensor	4
6	FS1	Focus Shaft	4
7	IN1	Brass Insert	4
8	FC1	Focus Cylinder	4
9	FA1	Focus Screw Assembly	4
10	DiF1	Dichroic Filter	4
11	ExF1	Excitation Filter	4
12	CL1	Condensor Lens	4
13	D1	Diffuser	4
14	L1	LED	4
15	HS1	Heat Sink	4
16	MH1	Middle Housing- LED casing	1
17	LC1	LED half Casing	4
18	MH2	Middle Housing	1
19	S1	Screw joint	4
20	IM	Implant sub-assembly	1

b

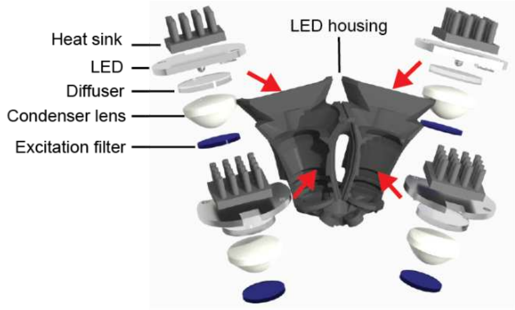

c

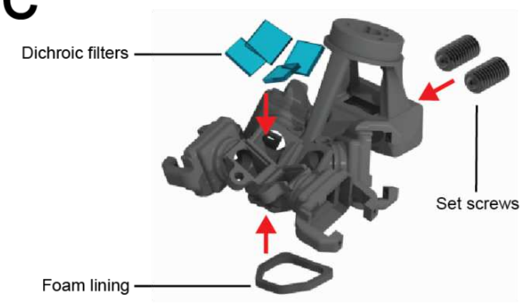

d

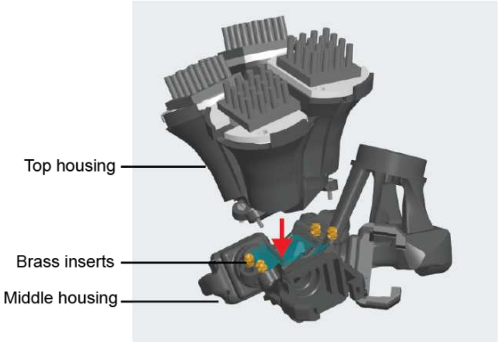

e

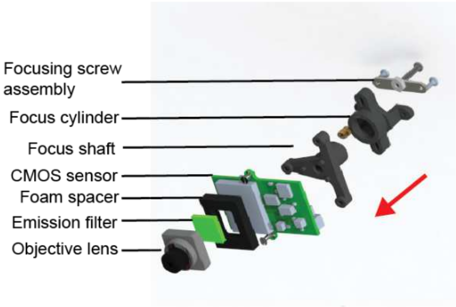

f

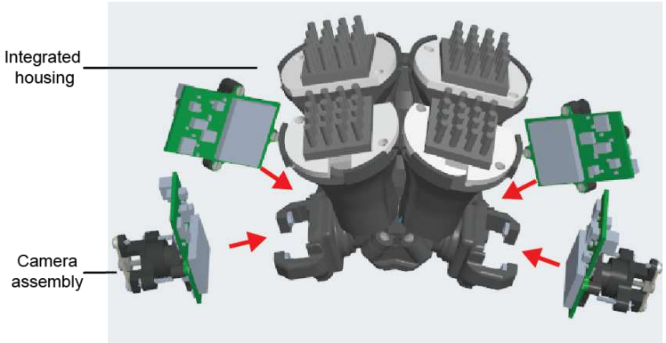

Supplementary Information 1: Additional CAD illustrations and assembly procedure for CortexCAM

a) An exploded CAD view of the CortexCAM assembly with unique part identifiers indicated in the CAD view and further detailed in the bill of materials table below.

b) A CAD view of the top excitation light module with each opto-electronic component highlighted. The red arrows indicate the assembly direction.

c) A CAD view of the middle housing sub-assembly where the red arrows indicate the assembly direction for the dichroics, setscrews and foam lining.

d) A CAD view to illustrate the assembly procedure for top and middle housing using four aligned holes fitted with brass inserts.

e) An exploded view of the camera assembly and the objective lens to show individual components and the red arrow shows the direction of assembly.

f) The final assembly step to attach the four camera sub-assemblies to the integrated CortexCAM housing. The red arrows indicate the assembly direction.

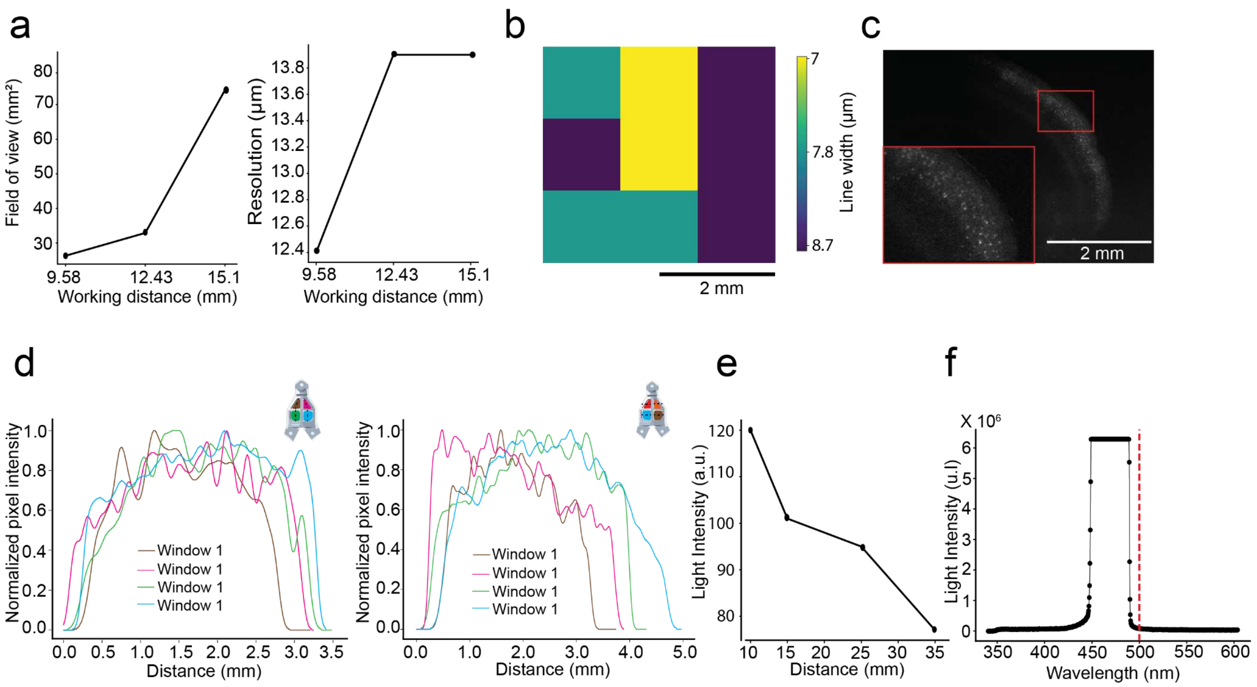

Supplementary Information 2: Additional benchtop optical characterization results for CortexCAM

a) Changes in FOV and resolution for the CortexCAM as a function of varying working distances obtained using a single camera module replica of CortexCAM

b) A Pseudo color heat map representing the variation in resolution across the field of view imaged using a single camera module replica of CortexCAM.

c) A 400μm coronal brain slice from a Cux2-CRE-ERT2 x Ai162 (TIT2L-GC6s-ICL-tTA2) imaged using a single camera module replica of the CortexCAM. The inset shows a zoomed in view of a sub-region that shows individual cell bodies present in layers 2 and 3 of the brain slice.

d) Light uniformity measurements made using a phantom implant, attached with a fluorescent tape on the bottom surface of the implant, across all four FOVs along the vertical and horizontal axes as shown in the respective inset figures.

e) Change in light intensity of the collimated light source module as a function of distance from the output aperture.

f) The measured light power spectrum for the collimated excitation light source with the red dashed line showing a cut off below 500nm.

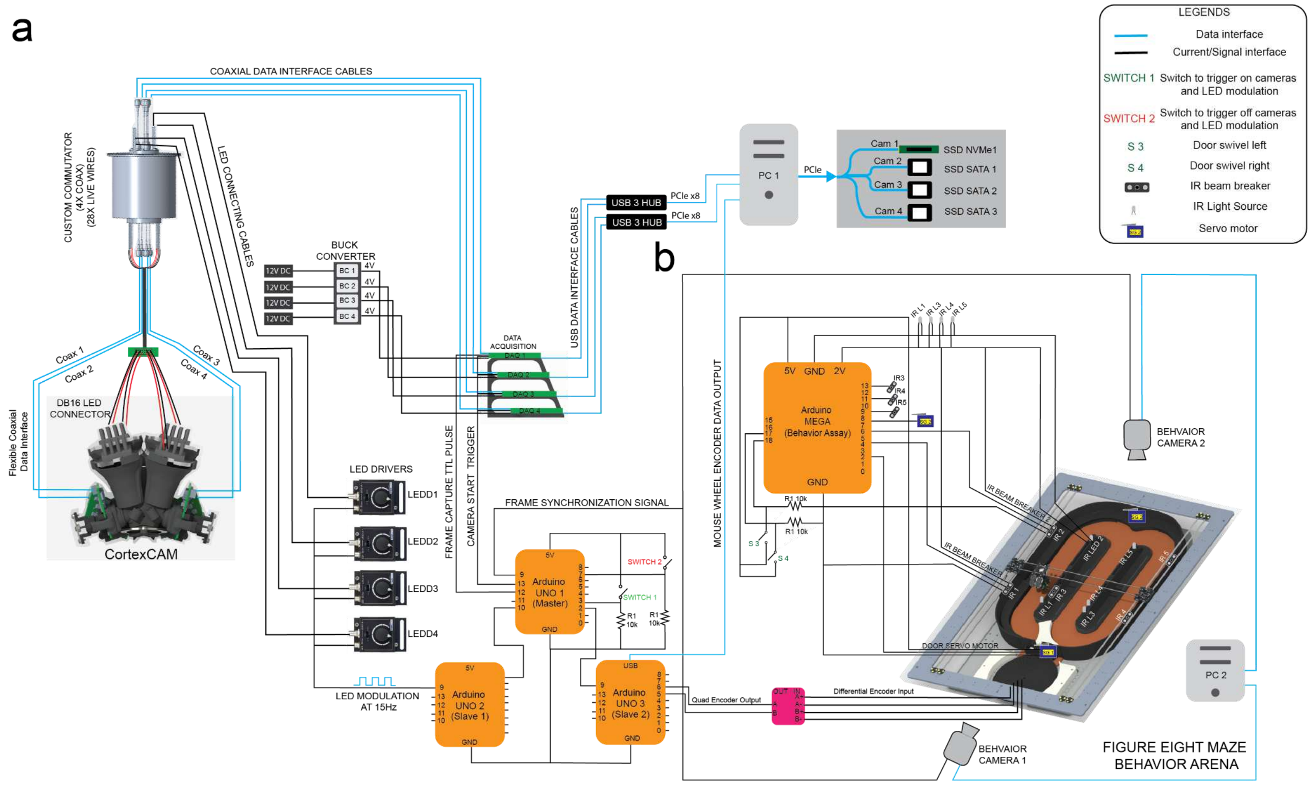

Supplementary Information 3: Mobile CortexCAM electrical wiring diagram

a) The wiring schematic for the CortexCAM device with blue connecting lines showing the data streaming, acquisition and storage connections and the black lines indicating the hardware automation, camera synchronization, LED modulation connections.

b) The wiring schematic for the automated figure eight maze behavior assay.

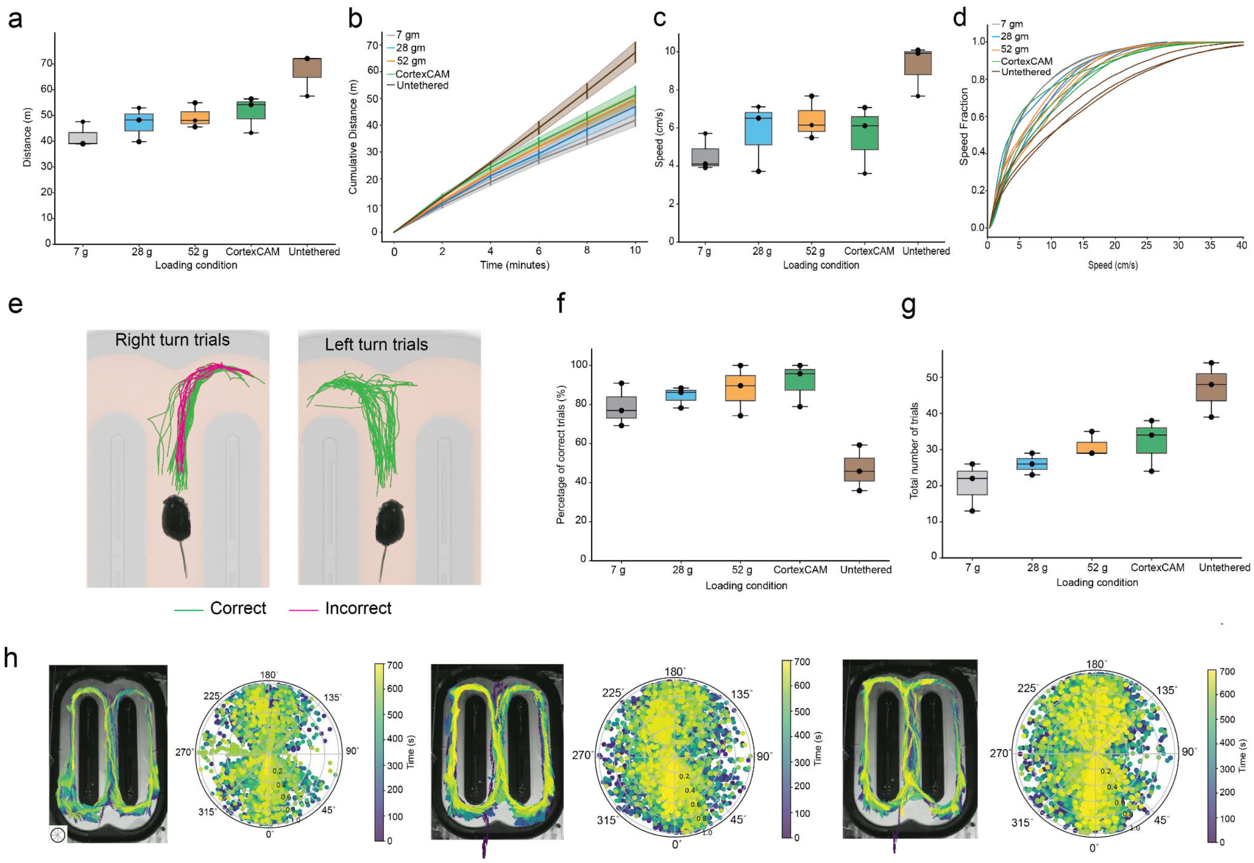

Supplementary Information 4: **Behavior data analysis of mice performing figure eight maze task.**

a) Distance moved by mice (n=3) across different loading conditions (Friedman test indicated that there is no statistically significant difference in distance moved by the subject between the conditions, χ^2 (4, N = 3) = 8.27, p = 0.08).

b) Cumulative distance moved by the mice (n=3) across 10 minute trial durations during different loading conditions.

c) Median speed distribution of mice (n=3) while performing a figure eight maze task during different loading conditions. (Friedman test showed no significant difference between conditions for speed of the subject, χ^2 (4, N = 3) = 7.73, p = 0.10).

d) The cumulative speed distribution of the mouse across different loading conditions while performing the figure eight maze task.

e)Trajectory of mice across all trials at the decision zone during the figure eight maze task. The correct trials are marked in green and the incorrect trials are marked as red.

f) Percentage of correct alternations by the mice (n=3) across different loading conditions. (The Friedman test showed statistical differences between conditions for the percentage of correct choices, χ^2 (4, N = 3) = 10.37, p = 0.03).

g) Total number of trials completed by mice (n=3) across different loading conditions.(Friedman test for the total number of trials performed during a 10 minute session showed no statistically significant differences between the conditions, χ^2 (4, N = 3) = 8.61, p = 0.07).

h) The trajectory and heading angle distribution of all three mice during a single trial performing the figure eight maze alternating choice task while tethered to the fully assembled CortexCAM.

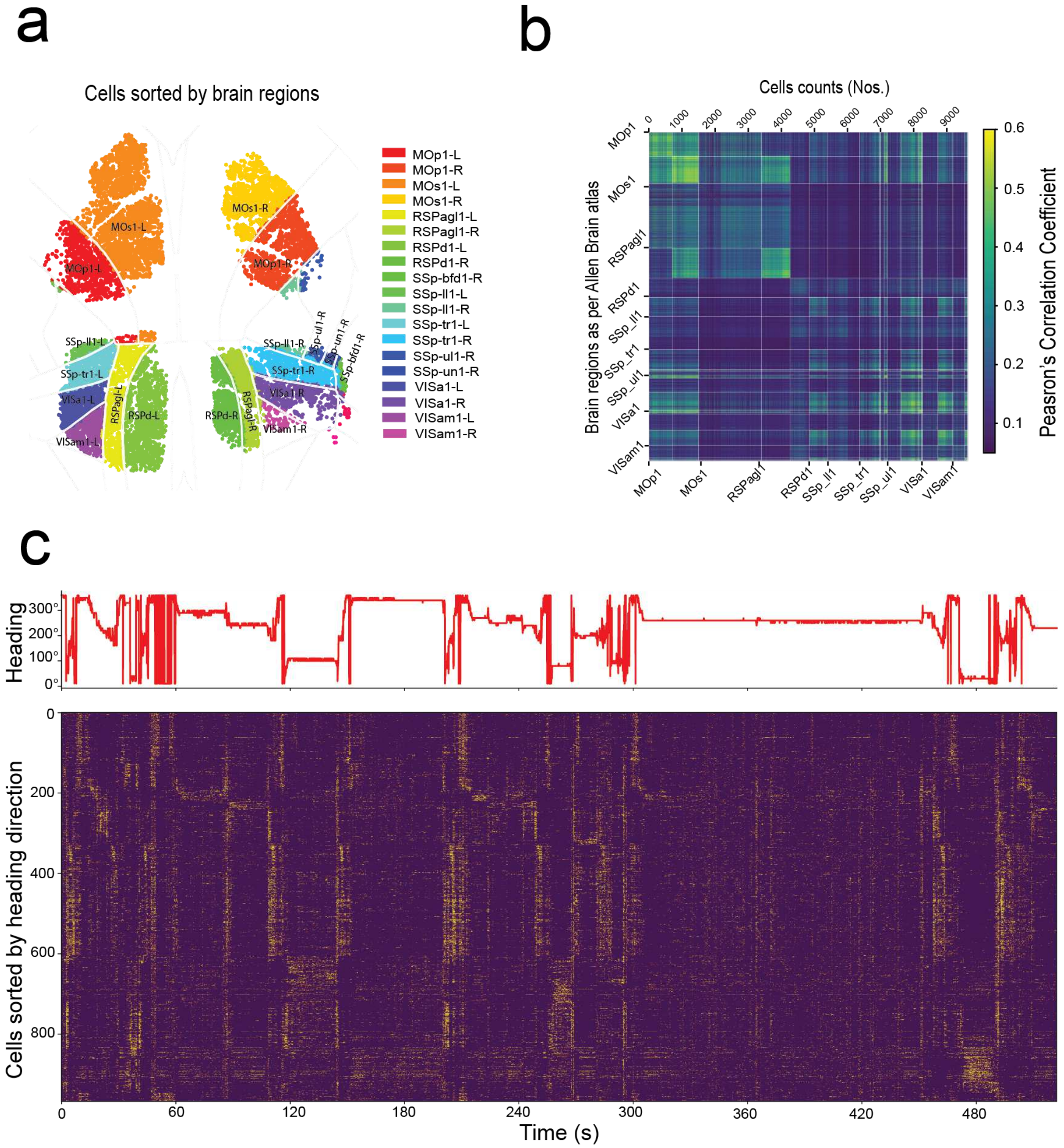
a
Cells sorted by brain regions
MOs1-L
MOp1-L
MOs1-R
MOp1-R
SSp-ll1-L
SSp-tr1-L
VISa1-L
VISam1-L
RSPagl-L
RSPd-L
SSp-ll1-R
SSp-tr1-R
SSp-ul1-R
SSp-un1-R
SSp-bfd1-R
VISa1-R
VISam1-R
RSPd-R
RSPagl-R
MOp1-L
MOp1-R
MOs1-L
MOs1-R
RSPagl1-L
RSPagl1-R
RSPd1-L
RSPd1-R
SSp-bfd1-R
SSp-ll1-L
SSp-ll1-R
SSp-tr1-L
SSp-tr1-R
SSp-ul1-R
SSp-un1-R
VISa1-L
VISa1-R
VISam1-L
VISam1-R
b
Cells counts (Nos.)
0 1000 2000 3000 4000 5000 6000 7000 8000 9000
Brain regions as per Allen Brain atlas
MOp1 MOs1 RSPagl1 RSPd1 SSp_ll1 SSp_tr1 SSp_ul1 VISa1 VISam1
Pearson's Correlation Coefficient
0.1 0.2 0.3 0.4 0.5 0.6
c
Heading
0° 100° 200° 300°
Cells sorted by heading direction
0 200 400 600 800
0 60 120 180 240 300 360 420 480
Time (s)

Supplementary Information 5: **Behavior data analysis of mice performing figure eight maze task.**

a) All cell regions detected using CortexCAM during a single imaging session while the mouse is freely locomoting. The cells are grouped by their anatomical brain regions as indicated by the color map.

b) A heatmap representing Pearson's correlation for the DFF activity of all cells shown in (a).

c) The heading direction (top) and sorted spike deconvolved trace activity (bottom) for the cells tuned to heading direction for the full trial as shown in figure 4.
